# Prevalent and Disseminated Recombinant and Wild-Type Adeno-Associated Virus Integration in Macaques and Humans

**DOI:** 10.1089/hum.2023.134

**Published:** 2023-11-15

**Authors:** Kelly M. Martins, Camilo Breton, Qi Zheng, Zhe Zhang, Caitlin Latshaw, Jenny A. Greig, James M. Wilson

**Affiliations:** Gene Therapy Program, Department of Medicine, Perelman School of Medicine, University of Pennsylvania, Philadelphia, Pennsylvania, USA.

**Keywords:** adeno-associated virus, gene therapy, human, next-generation sequencing, nonhuman primate

## Abstract

Integration of naturally occurring adeno-associated viruses (AAV; wild-type AAV [wtAAV]) and those used in gene therapy (recombinant AAV [rAAV]) into host genomic DNA has been documented for over two decades. Results from mouse and dog studies have raised concerns of insertional mutagenesis and clonal expansion following AAV exposure, particularly in the context of gene therapy. This study aimed to characterize the genomic location, abundance, and expansion of wtAAV and rAAV integrations in macaque and human genomes. Using an unbiased, next-generation sequencing-based approach, we identified the genome-wide integration loci in tissue samples (primarily liver) in 168 nonhuman primates (NHPs) and 85 humans naïve to rAAV exposure and 86 NHPs treated with rAAV in preclinical studies. Our results suggest that rAAV and wtAAV integrations exhibit similar, broad distribution patterns across species, with a higher frequency in genomic regions highly vulnerable to DNA damage or close to highly transcribed genes. rAAV exhibited a higher abundance of unique integration loci, whereas wtAAV integration loci were associated with greater clonal expansion. This expansive and detailed characterization of AAV integration in NHPs and humans provides key translational insights, with important implications for the safety of rAAV as a gene therapy vector.

## INTRODUCTION

Wild-type adeno-associated virus (wtAAV) is a helper-dependent, non-pathogenic parvovirus endemic to the human and nonhuman primate (NHP) populations. Recombinant AAV (rAAV) is the leading gene delivery vector because of its low immunogenicity, ability to transduce dividing and nondividing cells, and stable extrachromosomal transgene expression.^1,2^ Despite continued categorization as integration defective, researchers have documented a low frequency of (r)AAV integration into genomic DNA for over two decades.^3^ Cell line and murine studies have clearly established that wtAAV can establish a latent infection (in the absence of a helper virus) and integrate specifically at human chromosome 19q13.3-qter (*AAVS1*) in a viral Rep protein- and p5 promoter-dependent manner^1,4–7^; *AAVS1* orthologs are present on chromosome 19 in NHPs.^8^

However, *in vitro* studies using ligation-mediated (LM) polymerase chain reaction (PCR)-based methodologies to enrich AAV chromosomal junctions have also shown a low frequency of wtAAV integrations throughout the genome (*i.e.*, non-site-specific integrations).^9–17^ wtAAV is generally considered to lack significant pathogenicity in humans; however, conflicting recent studies suggest wtAAV may represent an extremely rare, but present, risk factor for hepatocellular carcinoma (HCC) development in humans.^18–24^ Despite its high prevalence in the human population, many elements of fundamental wtAAV biology *in vivo* in primates, including integration, remain poorly understood.

*Rep*-deficient rAAV sequences do not integrate at *AAVS1*, but integrations can still be observed at a low frequency throughout the host genome. The locations of rAAV integrations are largely considered to be random; several mouse and cell line studies have shown conflicting data on potential preferential rAAV integration locations within the (mammalian) genome.^10,25–27^ No common integration site has been reported in the limited number of *in vivo* investigations of rAAV integration in NHPs and humans following rAAV gene therapy administration.^28^ The distribution pattern of rAAV integration *in vivo* in primates has yet to be fully defined, and it is unknown whether rAAV integration is similar to non-site-specific wtAAV integration (*i.e.*, integration not occurring at *AAVS1*).

As the AAV gene therapy field progresses and more patients receive an increasing number of treatments, there is a growing need to characterize this therapeutic platform and its long-term consequences. Results from preclinical animal studies have highlighted the possibility of AAV gene therapy causing insertional mutagenesis (also referred to as genotoxicity). rAAV-mediated insertional mutagenesis was first reported in neonatal mice with integration events in the regulatory RNA-encoding *Rian* locus, which led to the development of HCC.^29^

Subsequent mouse studies have implicated factors such as newborn administration, strong promoter/enhancer elements, and specific mouse strain/vector combinations with the integration and development of HCC.^29–32^ Recent results from a long-term canine study have also raised concerns about clonal expansion of rAAV integration sites, although tumor formation has not been observed.^33^ While there is currently no report of insertional mutagenesis in any primate species or extrahepatic tissue of any species, the potential for oncogenicity due to rAAV integration warrants further investigation.

The primary aim of this study was to perform a large-scale, comprehensive, and unbiased characterization and direct comparison of rAAV and wtAAV integrations in primate genomes following *in vivo* exposure. We subjected tissues from 254 macaque and 85 human samples to the same method of detection (a modified version of our LM-PCR-based next-generation sequencing [NGS] technique termed “ITR-seq”^34^), analysis, and annotation. We characterized the locations of AAV integrations and assessed these locations for enrichment using integrative analyses of genomic, epigenomic, and transcriptomic data in primates. Representing the largest cohort comparison study in NHP and human samples in the literature to date, our large-scale direct analyses provide crucial insights with translationally relevant implications for current and future gene therapy studies and highlight the clinical translatability of NHP AAV integration data.

## MATERIALS AND METHODS

### Sample acquisition

#### Naïve NHP liver

We obtained livers from 168 male and female NHPs (cynomolgus [*Macaca fascicularis*] and rhesus [*Macaca mulatta*] macaques; <1 to >20 years old) naïve to rAAV exposure from primate reserves and facilities, including the Tulane National Primate Research Center, University of Puerto Rico Caribbean Primate Research Center, and two different facilities within the Mannheimer Foundation. Liver samples were flash-frozen before processing for DNA extraction.

#### AAV-exposed NHP liver

We obtained livers from 86 male and female NHPs (cynomolgus [*M. fascicularis*] and rhesus [*M. mulatta*] macaques; newborn to adult) treated with different preclinical gene therapy vectors from studies conducted within the University of Pennsylvania's Gene Therapy Program. These studies comprise rAAV investigations involving single-stranded AAV vectors with different liver-specific promoters/enhancers, transgenes, and capsid serotypes, all administered via intravenous injection across a range of doses (3 × 10^12^–5 × 10^13^ genome copies [GC]/kg) with post-rAAV treatment time points ranging from 7 days to 15 years (Supplementary Table S1).

#### wtAAV-exposed human liver

We obtained livers from 85 humans (male and female; 28 years old to >80 years old) from Origene (Rockville, MD) and BioIVT (Westbury, NY). The samples included healthy (25) and disease (45) state samples from individuals with varied past medical histories, causes of death, and sample histology (Supplementary Table S2).

### Sample preparation and modified ITR-seq library preparation

We modified our previously published “ITR-seq” method^34^ (designed to detect nuclease off-targets *in vivo*) to detect AAV integrations of all known AAV serotypes by using a pool of primers (Supplementary Fig. S1).

We isolated genomic DNA from a ∼10-mg piece of tissue from each liver sample using the MagMAX™-96 DNA Multi-Sample Kit (Invitrogen) or QIAGEN Genomic-tip DNA extraction kit. The genomic DNA was sheared into small fragments with an average size of 500 bp using an ME220 focused ultrasonicator (Covaris, Woburn, MA) followed by purification using AMPure beads (Beckman Coulter, Indianapolis, IN) at a 0.8 × ratio. Four hundred nanograms of purified DNA was then end-repaired (Enzymatics, Beverly, MA). Unique Illumina Y-adapters were ligated onto the sheared DNA containing a unique sample barcode sequence and unique molecular identifier (UMI). The UMI is an 8-bp sequence that labels each DNA fragment and enables the removal of PCR duplicates.

We amplified end-repaired, Y-adapter-ligated DNA by two rounds of PCR using equal concentrations of each of the three inverted terminal repeat (ITR) primers, in addition to adapter-specific primers that contain secondary barcode information. This PCR protocol was optimized to adequately denature the ordered secondary structure of integrated ITRs using a high annealing temperature (69°C) and uses longer adapter-specific primers and a pool of primers specific for conserved regions of all known AAV serotypes. We loaded the generated NGS-compatible dual-indexed libraries with amplicons containing both the amplified integrated AAV ITR and the adjacent genomic DNA sequences onto an Illumina MiSeq cartridge (MiSeq^®^ v2 RGT Kit 300 cyc PE-Bx 1 of 2; San Diego, CA), generating 2 × 150-bp paired-end reads. The subsequent FASTQ file of raw multiplexed base calls was subjected to bioinformatics analysis.

### Bioinformatics pipeline

We updated the previously published ITR-seq bioinformatics pipeline to process raw sequencing data, to computationally identify the location and frequency of genome-wide AAV integrations. Our bioinformatics analysis pipeline has been updated since its previous description^34^ to streamline steps, replace outdated programs, and allow the detection of integration site clones, individual unique rAAV integration loci in non-gene-editing studies, and wtAAV serotypes.

In summary, a config file with the sample information is created after sequence generation of the Illumina MiSeq-compatible libraries from the amplified DNA data. The demultiplexed sequencing files for each sample undergo the following steps: (1) UMI tagging of raw sequencing reads; (2) mapping to the indicated genome of interest; (3) identifying the genomic location of ITR–genome junctions (unique integration loci [UILs]); (4) removal of PCR duplicates using the UMI and read mapping information; and (5) annotation of the genomic location of each UIL.

The number of unique genome–AAV junctions was determined for each sample, and this number was normalized to 100 genomes based on input DNA. We determined the number of copies (expansions/clones) for each unique genome–AAV junction based on the number of reads at the same unique AAV–genome junction that contains unique adapter–genome junctions and unique UMIs. By requiring both a unique adapter position and a unique UMI for a given ITR integration position, we were able to differentiate between reads originating from cell clones (*i.e.*, same ITR position, but different adapter position and different UMI) and PCR duplicates (*i.e.*, same ITR position and same adapter position and/or same UMI) with an enhanced degree of accuracy over previous studies.

### Genomic distribution analyses

We computationally determined the genomic location of each unique AAV integration site from raw sequencing data for each sample. A unique AAV integration site represents a distinct integration event at a single base-pair genomic location directly adjacent to the AAV ITR sequence. The number of UILs was detected for each sample, and the number of distinct DNA copies was determined for that UIL. The number of clones represents the copy number of chimeric sequencing reads containing both a unique UMI and unique Y-adapter position for each UIL.

We mapped the integration loci for each sample to their respective host genome (human: GRCh38.p13; rhesus: MmuI_10; and cynomolgus: Macaca_fascicularis_5.0) using ChromoMap and Archive!Ensembl. The genomic location for each integration was annotated according to the host species RefSeqGene annotation. We annotated each AAV integration locus as being within (genic) or outside (intergenic) a gene-coding region. Integrations within genic regions were further annotated by location within an exon or intron and by expression level in the liver.

We determined expression levels through normalized expression (nx) in the human liver (or other tissues) for each annotated gene in the Human Protein Atlas (https://www.proteinatlas.org/). Categories were determined as follows: genes not expressed in the liver: 1 < nx; genes with low expression in the liver: 1 ≤ nx <10; genes with medium expression in the liver: 10 ≤ nx <100; and genes with high expression in the liver: nx ≥100. NA values represent a non-annotated genomic location.

We annotated each integration locus as being within a repeat region or outside of a repeat region (non-repeat region) according to the host genome RepeatMasker Annotation Track. AAV integration loci within repeat regions were further classified and compared according to family and class of repeat sequence.

We converted genomic coordinates from NHP genomes to the human genome (H.sapiens hg38) using the University of California, Santa Cruz liftOver tool. We then utilized the ChIP-Atlas (https://chip-atlas.org) to predict the proteins bound to integration loci. We performed enrichment analysis using all experiments in humans (H.sapiens hg38) in the liver cell-type class in the database for DNase sequencing (DNase-seq), assay for transposase-accessible chromatin using sequencing (ATAC-seq), chromatin immunoprecipitation (ChIP):transcription factors (TFs) and others, and ChIP:RNA polymerase. We also used the ChIPseeker R package to annotate coverage over chromosomes and profiles of peaks binding to transcription start site (TSS) regions.

We determined the percentage of integration loci corresponding to site-specific integrations for each sample. For each species, the coordinates used for each AAVS site are as follows: human (GRCh38.p13) *AAVS1*: chr19:53,100,001–55,800,000; *AAVS2*: chr5:28,900,001–33,800,000; and *AAVS3*: chr3:16300001–23800000; rhesus (MmuI_10) *AAVS1*: chr19:52871888–55563490; *AAVS2*: chr6:29000422–33933291; and *AAVS3*: chr2:23869443–31378156; cynomolgus (Macaca_fascicularis_5.0) *AAVS1*: chr19:53911962–56643542; *AAVS2*: chr6:29397402–34531455; and *AAVS3*: chr2:23678813–31274157.

### Randomly generated loci

We performed a comparison to a random distribution by generating 10,000 random loci for each species using the BedTools pseudo-random number generator and the number and length of chromosomes in the genome for each species.

### Statistical analyses

Unpaired *t*-tests were performed in quantitative comparisons between rAAV and wtAAV (number of UIL, proportion of loci clonally expanded, etc.). We performed standard one-way analysis of variance for multiple comparisons and distribution comparisons between rAAV and wtAAV groups, between each group and random loci, and between categories within each group. We also conducted additional *t*-tests with correction for multiple comparisons using RStudio.

### Data availability statement

All data discussed in the article are available in the main text or Supplementary Data files. ITR-seq data are available on GitHub (https://github.com/Penn-GTP/ITR-seq2_public, version 2.1.1). Complete clinical pathology data can be obtained upon request.

## RESULTS

### rAAV has a higher number of unique integration sites than wtAAV in NHPs

We subjected tissues from 86 NHPs treated with different rAAV-based preclinical gene therapies and 168 untreated NHPs to the same method of detection, analysis, and annotation. Both NHP groups comprised male and female, newborn to adult cynomolgus and rhesus macaques (Table 1). rAAV-administered NHPs received different preclinical gene therapy vectors with various liver-specific promoters/enhancers (thyroxin binding globulin [*TBG*] and non-*TBG*), transgenes (self, nonself, and human transgenes), capsid serotypes (including AAV8, AAVrh10, AAVhu37, and AAV3B), and varying doses (3 × 10^12^–1.2 × 10^13^ GC/kg), with time points ranging from 7 days to 15 years post-gene therapy treatment. Given the endemic nature of wtAAV infections in both humans and macaques, we expected to find integrated genomes in most rAAV-naïve samples following natural wtAAV infection upon normal environmental exposure.

**Table 1.** Sample information

AAV Exposure	Species	Health Status	Sex	Ages (Min and Max)	Time of AAV Exposure	No. of Primates Tested	No. of Primates with Detected Liver Integration	Total Liver UILs	Liver Site-Specific UILs (% of Total UILs)	Extrahepatic Tissue Tested
wtAAV	Cynomolgus and Rhesus Macaques	Healthy and diseased	Male and female	Newborn—Adult (0.5–30 years old)	Unknown	168	112	3,277	Yes (0.21)	Heart; Lung; Kidney; Brain; Spleen
wtAAV	Human	Healthy and diseased	Male and female	Adult (28–80 years old)	Unknown	85	51	1,836	Yes (1.14)	N/A
rAAV	Cynomolgus and Rhesus Macaques	Healthy	Male and female	Newborn—Adult (0.02–3+ years old)	7 days–15 years	86	86	61,685	Yes (0.06)	Heart; Lung; Kidney; Brain; Spleen

NHP samples were sourced from primate reserves and facilities naïve to rAAV exposure (wtAAV NHP), and human samples, also naïve to rAAV exposure (wtAAV human), were sourced from BioIVT and Origene and included 27 healthy and 58 diseased liver samples with varied past medical histories, causes of death, and sample histology (Supplementary Table S1). In diseased human patients, the liver samples were taken from a diseased section of tissue and included 14 hepatocellular carcinoma samples, as well as samples with fatty changes, portal inflammation, chronic inflammation, cirrhosis, congestion, steatosis, hepatitis, and hemosiderin deposition. Healthy patients did not have any clinical pathology, and the liver samples were confirmed to be histologically free from pathology. NHPs administered different preclinical gene therapies (rAAV NHP) at the Penn Gene Therapy Program included rAAV vectors with different promoters, enhancers, transgenes, capsid serotypes, and doses (Supplementary Table S2). Site-specific and non-site-specific integrations were detected in all chromosomes in all three groups. All samples were acquired at necropsy time points, and each NHP or human sample had liver tissue tested. In a small subset of NHPs (rAAV and wtAAV), extrahepatic tissues collected at necropsy were also tested.

AAV, adeno-associated virus; NHP, nonhuman primate; rAAV, recombinant AAV; UIL, unique integration locus; wtAAV, wild-type AAV.

We used an anchored multiplex PCR NGS-based technique for unbiased genome-wide detection and localization of UILs within each AAV-exposed (rAAV or wtAAV) NHP in our cohort. Adapters were ligated onto randomly sheared free ends of genomic DNA isolated from each liver sample. We amplified AAV DNA and adjacent genomic DNA at each UIL using a pool of primers that targeted ITRs (in rAAV vectors and in all known wtAAV serotypes) in combination with an adapter primer (Supplementary Fig. S1a, b). Samples were sequenced using the Illumina MiSeq paired-end platform, and UILs were localized within the host genome and were further characterized and annotated using a custom bioinformatics pipeline.

**Figure 1.**
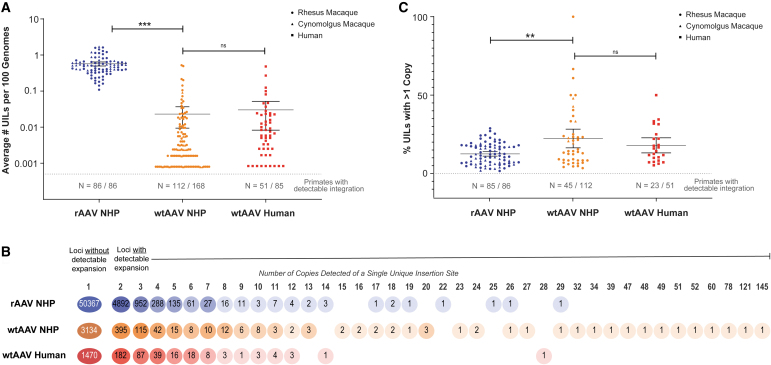
rAAV has a higher number of unique integration sites than wtAAV, but not a higher proportion of integration loci with detectable expansion. **(A)** The number of unique AAV integration sites for each sample was computationally determined from raw sequencing data using our bioinformatics pipeline. This number was normalized to the number of unique integration sites per 100 genomes based on the input number of genomes during library generation. The *dashed line* and *N* values listed below correspond to the number of samples with detectable integrations among the samples tested. The *circle* data points represent rhesus *Macaca mulatta* samples, the *triangle* data points represent cynomolgus *Macaca* samples, and the *squares* represent human samples. ***p < 0.001. **(B)** The number of copies was determined for each integration loci. The percentages of all rAAV NHP (*blue*), wtAAV NHP (*orange*), and wtAAV human (*red*) UILs with a given number of copies are shown as a heat map coloring. The number of all UILs with a given number of copies is listed within each *circle*. **(C)** The percentage of integration loci with more than one copy detected was determined for each sample. The *dashed line* and *N* values listed below correspond to the number of samples with detectable integration, which also had detectable expansion. The *circle* data points represent rhesus *M. mulatta* samples, the *triangle* data points represent cynomolgus *Macaca* samples, and the *squares* represent human samples. **p < 0.01. NHP, nonhuman primate; ns, nonsignificant; rAAV, recombinant adeno-associated virus; UIL, unique integration locus; wtAAV, wild-type adeno-associated virus.

We detected rAAV integrations in all 86 NHPs tested. We observed wtAAV integrations in 112 of the 168 naive NHPs sourced from primate facilities, reserves, and research centers in the United States and South America (Table 1). The average number of UILs detected in rAAV NHPs was significantly higher than that in wtAAV NHPs (rAAV: 0.57 UILs per 100 genomes and wtAAV: 0.02 UILs per 100 genomes; *p* < 0.0001) (Fig. 1a). The number of UILs detected in each NHP was widespread, with high variability most notable among wtAAV NHPs (coefficient of variation for rAAV: 56.1%; wtAAV: 318%).

To evaluate variables potentially affecting rAAV-associated integration, we normalized all samples to a dose of 1 × 10^13^ GC/kg for comparison. The UIL number did not significantly differ based on necropsy time point (7 days to 15 years) (Supplementary Fig. S2a), age of NHP at injection (newborn vs. adult), transgene (non-self vs. human vs. self), or promoter (*TBG* vs. non-*TBG*) (Supplementary Fig. S2b). We analyzed paired samples from 10 NHPs comprising an early biopsy (14–336 days) along with a later necropsy (760–1093 days) to determine the UIL number over time; 9/10 had fewer UILs at necropsy than at the prior biopsy (Supplementary Fig. S2c).

### wtAAV has a higher proportion of integration loci with detectable clonal expansion than rAAV in NHPs

Detection of more than one copy of the same UIL within the same animal indicates expansion of the cell in which the initial insertion occurred. During library preparation, we ligated Y-adapters with UMIs onto randomly sheared genomic DNA free ends before performing PCR to help detect and quantify expansions (Supplementary Fig. S1a). We defined clonal expansion as two or more distinct copies of the same UIL, with each copy required to have both an independent UMI sequence and an independent location of the ligated adapter.

The number of copies detected for each UIL was low in both rAAV and wtAAV NHPs (average of 1.1 copies/rAAV UIL and 1.8 copies/wtAAV UIL; both had a mode and median of 1 copy/UIL) (Fig. 1b). We detected expansion (2 or more copies detected for a single UIL) in 6,980 of the total 61,685 rAAV UILs (11.3%) and in 550 of the total 3,277 wtAAV NHP UILs (16.8%). At loci with detectable expansion, the average number of copies was higher in wtAAV NHPs than in rAAV NHPs (average of 2.4 copies/rAAV clonal UIL vs. 3.3 copies/wtAAV clonal UIL; both had a mode and median of 2 copies/clonal UIL) (Fig. 1b).

The highest detectable expansion of a single UIL was 29 copies in rAAV NHPs and 145 copies in wtAAV NHPs (Fig. 1b). In each NHP with detectable expansion, wtAAV also had significantly higher levels of total UILs with detectable expansion (average of 12.5% rAAV vs. 22.3% wtAAV; *p* = 0.0002) (Fig. 1c). wtAAV also had higher levels of variation among NHPs with detectable expansion (coefficient of variation for rAAV:15.6%; wtAAV: 89.7%).

In rAAV NHPs, the UIL number did not increase as a factor of time (Supplementary Fig. S2a); however, the UIL expansion did (Supplementary Fig. S2d). As the time between injection and necropsy increased, the number of copies detected at clonal loci also increased. Specifically, in samples collected at earlier necropsy time points, the majority of clonal loci was limited to two copies. However, in samples obtained at later necropsy time points, there was a notable rise in the number of clonal loci with 3–5 or 6+ copies, along with a decrease in loci with 2 copies. This trend was particularly pronounced in newborn injected NHPs (Supplementary Fig. S2d).

Clonal loci at early time points following rAAV exposure primarily had 2 detectable copies; at later time points, clonal loci with 2 copies decreased and loci with 3–5 or 6+ copies increased, with a more pronounced effect in newborn-injected NHPs (Supplementary Fig. S2d).

### wtAAV and rAAV integration loci have similar, broad distributions throughout the NHP genome

The distribution of wtAAV and rAAV UILs followed broadly similar patterns throughout the autosomal and sex chromosomes in both cynomolgus and rhesus macaques (*p* = 0.40 and *p* = 0.83, respectively) (Supplementary Figs. S3 and S4). When the number of UILs was normalized to chromosome size, both rAAV and wtAAV exhibited a random distribution pattern related to the chromosomal size (within 1-fold change), with the exceptions of rhesus wtAAV (2.2-fold higher in chromosome 19) and cynomolgus wtAAV (3.2-fold lower in chromosome 3), and 1- to 2-fold decreases in the sex chromosomes (Supplementary Figs. S3 and S4).

**Figure 2.**
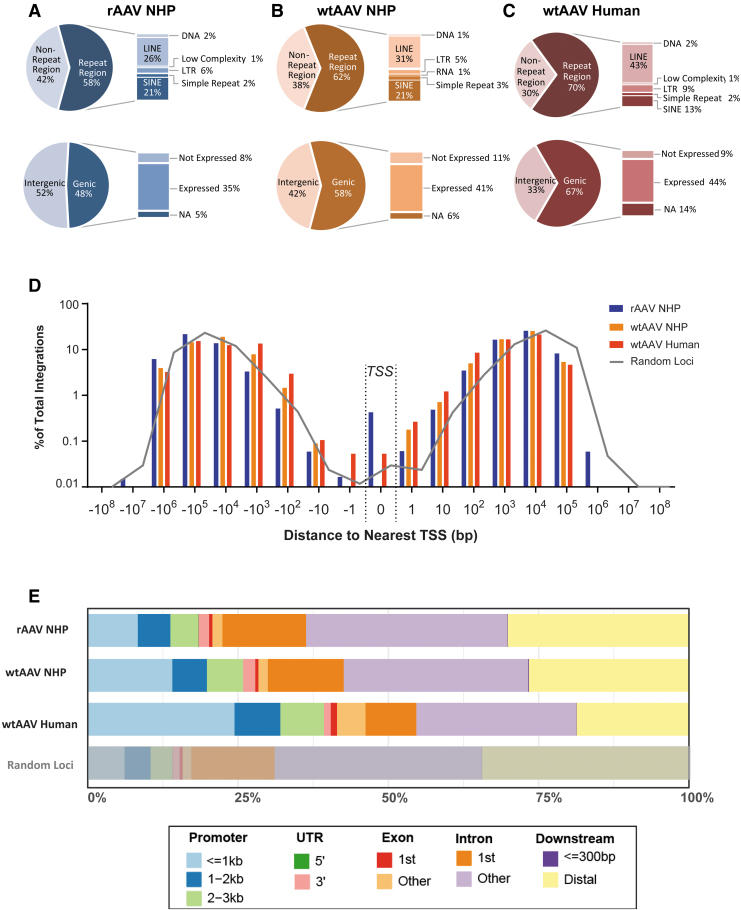
wtAAV and rAAV integration loci have similar broad distributions throughout the NHP and human genomes. **(A–C)** The genomic location for each integration was annotated as being within a gene-coding region (genic) or outside of a gene-coding region (intergenic) according to the given species' RefSeqGene annotation. Integrations within genic regions were further annotated by their respective expression level in the liver. The genomic locations for each integration were also annotated as within a repeat region or outside of a repeat region (non-repeat region) according to the given species' RepeatMasker Annotation Track. **(D)** Each integration locus was annotated for the distance to the nearest gene's TSS. The results are graphed as a histogram according to the percentage of total loci between each designated bin. Bin values are listed as the distance (in base pairs) from the TSS, where 0 represents the exact base pair of a gene's TSS. Negative values indicate that the insertion locus is downstream of the nearest TSS, whereas positive values indicate that the locus is upstream of the nearest TSS. The *gray line* indicates the distribution of randomly generated loci and the distribution of distance to the nearest gene's TSS. **(E)** The genomic location for each integration site was annotated according to location within promoter, UTR, exon, intron, or downstream intergenic regions. Percentages indicate the proportion of loci within that region. LINE, long interspersed nuclear element; LTR, long terminal repeat; NA, not annotated; SINE, short interspersed nuclear element; TSS, transcription start site; UTR, untranslated region.

To provide context about the location of integration sites, we annotated the genomic location of each UIL using RefSeqGene and RepeatMasker Annotation Tracks; the integration site patterns were again determined to be similar between rAAV and wtAAV (Fig. 2a, b and Supplementary Fig. S5a, b). rAAV and wtAAV exhibited similar proportions of UILs within genic and intergenic regions and within repeat and non-repeat regions (Fig. 2a, b). The distribution of UILs in relationship to the TSS of the nearest gene was very similar for wtAAV and rAAV (*p* = 0.9998) (Fig. 2d). The vast majority of rAAV and wtAAV genic UILs was located within introns and within genes expressed in the liver (Fig. 2a, b), whereas intergenic integrations were evenly distributed between proximal (promoter/untranslated region) and distal regions (Fig. 2e).

### In NHPs, rAAV and wtAAV integration loci are enriched in highly transcribed genomic regions and those vulnerable to DNA damage

To determine whether rAAV or wtAAV displays preferential sites of integration within the genome, we compared the proportion and distribution pattern of rAAV and wtAAV UILs with 10,000 random genomic loci for each NHP species. wtAAV and rAAV UILs did not significantly differ from the distribution of random loci within intergenic, genic, repeat, or non-repeat regions, except in highly transcribed and/or open chromatin regions.

**Figure 3.**
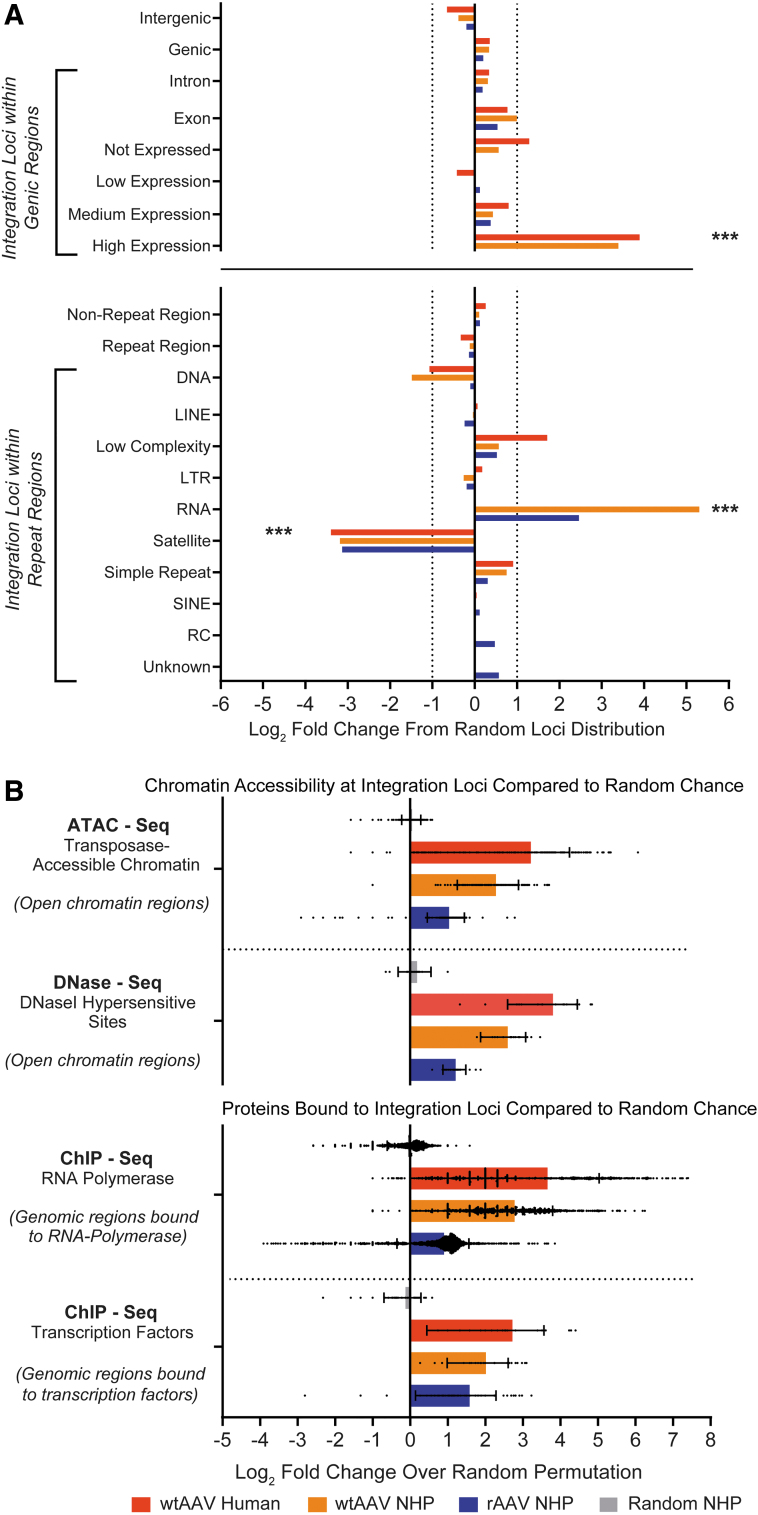
rAAV and wtAAV integration loci exhibit a non-random increase within genomic regions highly vulnerable to DNA damage. **(A)** The genomic location for each integration was annotated as being within a gene-coding region (genic) or outside of a gene-coding region (intergenic) according to the given species' RefSeqGene annotation. Genic integrations were annotated according to their respective expression level in the liver. The genomic locations for each integration were also annotated as being within a repeat region or outside of a repeat region (non-repeat region) according to the given species' RepeatMasker Annotation Track. Comparison to a random distribution was performed for each species by taking 10,000 randomly generated genomic loci for each species and comparing them with the AAV integrations from each group. The fold change from the random distribution was determined, in which 0 represents the random distribution, a positive fold change indicates an increase in integration loci in the region, and a negative fold change indicates a decrease. AAV integrations within repeat regions were compared based on the class of repeat: DNA: DNA transposon; LINE; Low Complexity: low-complexity DNA; LTR; RNA: RNA repeats (rRNA, RNA, transfer RNA, small nuclear RNA, small conditional RNA, signal recognition particle RNA); Satellite: satellite repeats; Simple Repeat: single-nucleotide stretches or tandem repeats; SINE; RC: other repeats (including *rolling circles*); Unknown: unknown repeat classification. ***p < 0.001. **(B)** We used the ChIP-Atlas database (https://chip-atlas.org) to determine the predicted proteins bound to genomic regions containing integration loci. For each experiment, the number of peaks that overlap with AAV integration loci was compared with overlap from a highly randomized background generated by a 100 × permutation model (each UIL in a dataset was permutated on a random chromosome at a random position 100 times). The fold change was determined, where 0 indicates no enrichment compared with the random loci. A randomly generated set of 10,000 loci was also subjected to the same analysis, as indicated in *gray*. ATAC, assay for transposase-accessible chromatin; ChIP, chromatin immunoprecipitation; rRNA, ribosomal RNA.

There was a non-random enrichment in genic regions highly expressed (nx >100) in the liver, with a 4.1-fold increase in rAAV UILs and 10.5-fold increase in wtAAV UILs; in RNA repeat regions, with a 5.5-fold increase in rAAV and 39.5-fold increase in wtAAV; and in satellite repeat regions, with an 8.8-fold decrease in rAAV and 9.1-fold decrease in wtAAV (Fig. 3a). The only RNA subclass that differed significantly from the random loci was ribosomal RNA (rRNA) repeats, which correspond to a region of high transcription.

To determine whether the genomic locations of UILs displayed preferential sites of integration within regions with epigenome modifications, we compared both the wtAAV and rAAV UIL datasets with a highly randomized background generated by a 100 × permutation model (each UIL in a dataset was permutated on a random chromosome at a random position 100 times). We aligned our identified AAV UILs against the ChIP-Atlas database,^35^ assessing for enrichment over a random background within regions with increased chromatin accessibility and open chromatin conformations (as determined by ATAC-seq and DNase-seq) and regions that directly bind TFs or RNA polymerase II (PolII) (as determined by ChIP sequencing [ChIP-seq]), all of which are factors that increase susceptibility to DNA damage (Fig. 3b).

The proportion of rAAV and wtAAV loci that overlapped with regions with open chromatin structure was significantly enriched in the DNase-seq and ATAC-seq datasets when compared with random loci (rAAV was enriched in 23/23 DNase-seq and 237/256 ATAC-seq datasets, with an average 2.1- to 2.3-fold increase; wtAAV was enriched in 16/18 DNase-seq and 196/197 ATAC-seq datasets, with an average 4.9- to 6-fold increase) (Fig. 3b). The proportion of rAAV and wtAAV loci that overlapped with genomic regions that directly bind TFs and RNA PolII was also significantly enriched in the ChIP-seq datasets when compared with random loci (rAAV was enriched in 1596/1900 TF and 71/74 RNA PolII datasets, with an average 1.9- to 3-fold increase; wtAAV was enriched in 33/33 RNA PolII and 1346/1355 TF datasets, with an average 4- to 6.9-fold increase) (Fig. 3b).

### Biology of wtAAV integrations in humans is similar to that observed for rAAV and wtAAV integrations in NHPs

To determine whether the observed patterns are unique to NHPs, we analyzed healthy and diseased human liver samples from 85 rAAV-naïve individuals (Table 1). Our human cohort consisted of tumor samples from 15 patients with HCC, diseased tissue samples from 44 non-HCC patients (fatty changes, congestion, portal inflammation, hepatitis, cirrhosis, cholestasis, hemosiderin deposition, and steatosis), and normal liver samples from 26 healthy patients. We detected a total of 1,836 UILs in the 85 human samples, in which 51 individuals (60%) had at least one detectable wtAAV integration (52% of normal samples, 66% of diseased samples, and 57% of tumor samples) (Supplementary Table S1).

There was no discernible difference between NHPs and humans in the detected number of wtAAV UILs per 100 genomes (0.030 wtAAV UILs/100 human genomes; *p* = 0.70) (Fig. 1a). Of the 1,836 total human wtAAV UILs, only 366 had detectable expansion of two or more copies. Samples with detectable expansion had an average of 18.3% UILs expanded (Fig. 1c), which was not different from wtAAV NHPs (*p* = 0.41). The average expansion was 3.3 copies/clonal UIL, nearly identical to our finding for wtAAV NHPs, with a maximum detectable expansion of 28 copies, which was detected in one healthy sample (Fig. 1b).

We observed similar broad distribution patterns across all chromosomes in the NHP and human genomes, with a similar increase in chromosome 19 (*i.e*., the chromosome with the most protein-coding genes), an additional decrease in chromosome 13 (a chromosome with low gene density) (Supplementary Fig. S6), and a nearly identical distribution of distance to the nearest TSS (Fig. 2d and Supplementary Fig. S5a, c). The human and NHP UILs followed the same distribution pattern among genic, intergenic, and repeat annotated regions (Fig. 2c), with a modest increase in genic regions with expression and within promoter regions (Fig. 2c, e). Human UILs were enriched an average of 14.9-fold in genes highly expressed in the liver (nx >100) (Fig. 3a), 7.7- and 15.4-fold in regions in which RNA PolII and TFs bind, and 10.1- to 12.8-fold in regions with open chromatin (Fig. 3b).

We observed site-specific integrations in all three test groups, identified within site-specific genomic locations (*AAVS1*, *AAVS2*, and *AAVS3*) in the human genome or the macaque genome ortholog (Supplementary Fig. S7). For both wtAAV NHPs and humans with site-specific integrations detected, they accounted for an average of 7.2% of UILs. Most rAAV NHPs had at least one detectable UIL identified within site-specific locations (71/86 NHPs); however, these UILs only represented a small minority of all rAAV UILs (0.5% of rAAV UILs were site specific) (Supplementary Fig. S7).

### The biology of rAAV and wtAAV integrations in extrahepatic tissues is comparable to the integration observed in the liver

To expand our investigation beyond the liver, we also analyzed heart (9 rAAV; 8 wtAAV), kidney (12 rAAV; 12 wtAAV), lung (11 rAAV), spleen (6 rAAV; 14 wtAAV), and brain (6 rAAV) tissues from 13 of the rAAV and 18 of the wtAAV rhesus macaques within our cohort and compared our findings with the matched NHP liver data.

**Figure 4.**
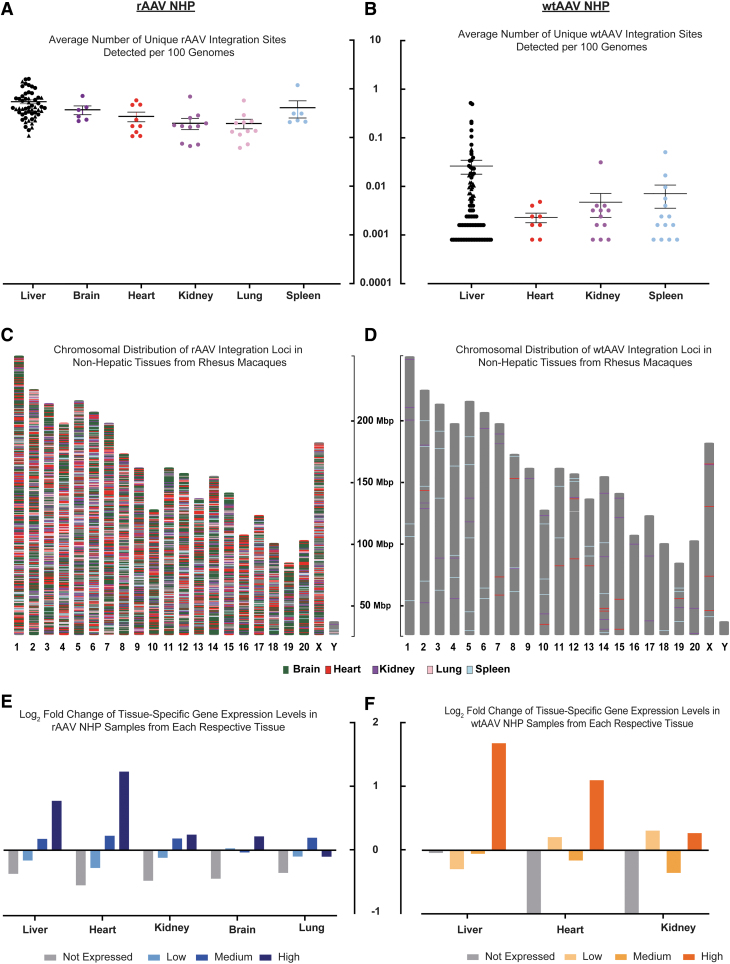
Quantitative and qualitative observations of rAAV and wtAAV integration loci in macaques are not limited to hepatic tissue. **(A, B)** Number of unique AAV integration sites detected in different tissues from (A) rAAV-treated rhesus NHPs and **(B)** wtAAV rhesus NHPs. This number was normalized to the number of unique integration sites per 100 genomes based on the input number of genomes during library generation. The liver results include all NHPs from our cohort, including the NHPs with other tissues studied. **(C, D)** Chromosomal graph showing the locations in the rhesus genome of UILs detected in tissue samples from **(C)** rAAV-treated NHPs and **(D)** wtAAV NHPs. The chromosome color is *gray*, and color-coded lines indicate locations of UILs according to the tissue in which they were detected. **(E, F)** For liver tissue samples from the 13 rAAV **(E)** and 18 wtAAV **(F)** rhesus with multiple tissues, the integration loci were annotated for their location within genic regions. Expression levels were determined by nx in the human liver for each annotated gene. Categories were determined as follows: genes not expressed: 1 < nx; genes with low expression: 1 ≤ nx <10; genes with medium expression: 10 ≤ nx <100; genes with high expression: 100 ≤ nx. Each value was compared with that of a random distribution, and the log_2_-fold change from the random distribution was plotted. nx, normalized expression.

Liver samples from rAAV and wtAAV NHPs had a higher average number of detected UILs than the other tissue samples (liver: rAAV 0.55 and wtAAV 0.03 loci/100 genomes; heart: rAAV 0.27 and wtAAV 0.002 loci/100 genomes; kidney: rAAV 0.20 and wtAAV 0.005 loci/100 genomes; spleen: rAAV 0.41 and wtAAV 0.007 loci/100 genomes; lung: rAAV 0.19 loci/100 genomes; and brain: rAAV 0.37 loci/100 genomes) (Fig. 4a, b). The UILs in extrahepatic tissues were broadly distributed throughout all the chromosomes (Fig. 4c, d), with a tissue-specific distribution in genic regions dependent on the respective tissue's expression profile. As observed in the liver samples, there was an enrichment of UILs within genes highly expressed in the respective tissue, whereas integration into genes not expressed in the respective tissue decreased (Fig. 4e, f).

## DISCUSSION

We performed a large-scale, comprehensive, unbiased study to characterize rAAV and wtAAV integration in NHP and human genomes. Our data do not suggest there is a higher risk of locational genotoxicity or expansion of rAAV integrations compared with natural wtAAV integrations in humans and macaques, thereby providing important insights into AAV integration that can inform preclinical and clinical genotoxicity risk assessment in a gene therapy context.

Our results support the notion that exposure to wtAAV or rAAV carries a similar chance for integration at a given non-*AAVS1* location in the primate genome, but that treatment with rAAV results in a higher number of integration loci. We observed a non-random increase in integration loci within highly expressed genic regions and in rRNA repetitive regions, which are highly transcriptionally active and/or susceptible to DNA damage.^27,28,36–38^ This finding was supported by ATAC-seq, DNase-seq, and ChIP-seq data in which UILs were enriched in genomic regions with open chromatin confirmations and that directly bind transcriptional machinery. Previous studies have shown increased rAAV integration in the presence of DNA damage, likely through induced chromosomal DNA double-stranded breaks (DSBs).^34,37–39^ These studies also suggest that the mechanism of rAAV integration is similar for induced and spontaneous DSBs based on similar microhomologies, insertions, and deletions around the integration sites.^37,38^

Our results indicate that non-site-specific wtAAV and rAAV integrations occur more frequently in locations of DNA damage and likely occur through a similar, passive process involving non-homologous end-joining repair of spontaneous chromosomal DSBs. Mammalian cells have endogenous enzymes that can efficiently ligate two DNA sequences regardless of end sequence homology^40^; thus, exogenous DNA delivered by any method may carry similar risks of integration. However, DNA sequences with repeats, origins of replication, and bent structures can influence integration and induce chromosomal instability.^40–47^ As wtAAVs and rAAVs contain ITRs that could form hairpin structures and contain origins of replication, the possibility of active integration cannot be dismissed.

Prevailing data indicate that rAAVs are safe and effective gene delivery vectors for numerous broad clinical indications. Of the serious adverse outcomes in the >200 rAAV-based clinical studies conducted to date, none have yet been attributed to rAAV integration.^48^ However, various preclinical studies suggest that AAVs can cause insertional mutagenesis leading to malignant transformation. Retrospective analyses of mouse studies indicate an increased risk of genotoxicity if rAAV treatment occurs during the neonatal period and/or involves high doses (1 × 10^14^ GC/kg) of rAAV containing strong promoter-enhancer elements.^49–51^

Our cohort of rAAV-treated NHPs spans a broad range of injection age (newborn to adult), time of necropsy post-rAAV exposure (7 days to 15 years), vector elements, and doses. Our analyses did not indicate that age at injection or the use of strong promoter-enhancer elements impacted the number of UILs detected in NHPs, although these analyses may not reflect the potential impact of these factors on the risk of malignant transformation following integration.

Long-term studies of rAAV-treated canines have not exhibited insertional mutagenesis or tumor formation, but have provided evidence of clonal expansion of integration sites. Within our rAAV-treated NHP cohort, only a small proportion of insertion sites had detectable clonal expansion, and these sites were distributed throughout the genome. Our largest detected expansion was 29 copies of a single UIL, which is relatively modest compared with recent canine studies^52^ and the wtAAV integrations observed within our cohort, the highest of which was 145 copies. Our data suggest that the UIL number does not increase over time, although the number of copies (clones) might.

This trend was apparent in the paired samples observed over time (at biopsy and then subsequent necropsy), especially those from newborn rAAV-treated NHPs. This pattern was likely due to developmental organ growth, normal cellular division/turnover, or repair following AAV toxicity-related tissue injury. wtAAV integrations and copy numbers may be markers for hepatocyte division and repopulation in the setting of health and disease. The lack of causal association between cancer and wtAAV remains reassuring, especially given the endemic nature of wtAAV in the human population. Detailed analysis of factors influencing integration and clonal expansion (and their potential consequences) is warranted, but beyond the scope of this study.

The incorporation of gene therapy into the standard of care for some diseases, including the approval of AAV products for hemophilia A and B, compels us to develop (standardized) methods for characterizing, detecting, and monitoring phenomena with potential health consequences, such as rAAV integration. The modified ITR-seq method used in this study to detect and characterize integrations is powerful and sensitive. However, it is possible that not all wild-type integrations were captured, as the isolation of ITR sequences has not been carried out for all identified wtAAV serotypes. In addition, rAAVs or wtAAVs that have concatemeric, rearranged, and/or partially deleted integrated genomes may not have been captured due to a potential lack of intact ITR sequences. Alternative approaches using long-read sequencing, DNA barcoding, and alternative multiplexed PCR-based techniques should be considered for detecting and characterizing the implications of AAV integration and expansion.

The current follow-up time for patients receiving gene therapy products is 5 years, which is supported by our cross-sectional and longitudinal studies. Our data comprised NHP liver samples up to 15 years post-rAAV treatment and indicate that the UIL number does not increase, and may decrease, over time. This finding suggests that integration in potentially genotoxic sites could be detected early after administration to determine an individual's risk, which would likely not change over the long term. While current data suggest that, in primates, rAAV administration/exposure is not associated with insertional mutagenesis with oncogenic potential, collecting any/all data to comprehensively characterize the effects of this relatively nascent therapeutic platform is warranted and appropriate.

The scarcity of human biopsy samples from gene therapy patients in target organs such as the liver, brain, and heart currently limits our ability to study rAAV integration in a clinical setting. However, the striking qualitative and quantitative similarity between our naïve NHP and human cohorts emphasize the translatability of AAV integration studies performed in NHP models and support their use in risk assessment studies over other biologically divergent preclinical animal models. For example, investigation of site-specific integrations in the human genome has been previously limited to cell line studies.

The initial studies that indicated preferential wtAAV integration at *AAVS1* were performed in cultured HeLa cells, a highly proliferative carcinoma-derived cell line harboring significant chromosomal rearrangements (up to 45% of integrations in *AAVS1*). Later studies utilizing diploid human fibroblast cell lines found that *AAVS1* was only targeted by 2.5% of all integrations. In combination with our observations in our wtAAV human cohort, these results suggest that *in vitro* transfection of proliferative cell lines induces a different insertional profile compared to what occurs during natural AAV infection in humans. In addition, our data support the use of samples from the large, easily accessible wtAAV-infected population (NHPs and humans) to expedite the development and validation of rAAV integration detection and monitoring tools for clinical practice.

The fact that rAAV genomes do integrate at relatively high frequencies, which can lead to HCC in high-dose newborn mouse studies, raises concerns about genotoxicity in human gene therapy studies.^31,48^ We are unaware of large-animal or human data implicating an rAAV or wtAAV integration in the development of a malignancy. Our ability to evaluate the expansion of integrations, which is likely a harbinger of cancer, can provide information regarding the oncogenic potential of AAV products as a platform and/or as individual AAV products. We were reassured to find very little clonal expansion in NHPs exposed to rAAV or wtAAV. Similarities in the biology of AAV integrations between humans and NHPs and the lack of an association between HCC and wtAAV infections in humans suggest a low risk of vector-induced malignancies in human gene therapy recipients. However, continued vigilance is warranted in monitoring gene therapy recipients for genotoxicity.

## Supplementary Material

Supplemental data

Supplemental data

Supplemental data

Supplemental data

Supplemental data

Supplemental data

Supplemental data

Supplemental data

Supplemental data

Supplemental data
