# Supplementary material for: Prevalent and Disseminated Recombinant and Wild-Type Adeno-Associated Virus Integration in Macaques and Humans
Source: Hum Gene Ther. 2023 Nov 15;34(21-22):1081–94. doi: 10.1089/hum.2023.134 (PMC10659022; doi:10.1089/hum.2023.134)
Supplement: Supplemental data [file Supp_TableS1.docx]

**Supplemental Tables**

**Table S1: Details of recombinant adeno-associated virus (rAAV) vector treatments given to the rAAV-treated nonhuman primates (NHPs)**

| **Capsid Serotype** | **Transgene** | **Promoter** | **Age at Injection** | **Necropsy Timepoint** | **Number of NHPs** |
| --- | --- | --- | --- | --- | --- |
| AAV2 | rhEPO | RSV | Adult | 15 years | 1 ^a^ |
| AAV3B | hFVIII | TBG-S1 | Adult | 3 Years | 2 |
|  | rhAFP | TBG | Adult | 6 Months | 3 |
|  | rhCG | TBG | Adult | 6 Months | 3 |
| AAV3B-8VR9 | hFVIII | TBG-S1 | Adult | 6 Months | 2 |
| AAV5 | rhAFP | TBG | Adult | 6 Months | 3 |
|  | rhCG | TBG | Adult | 6 Months | 3 |
| AAV8 | eGFP | TBG | Adult | 7 Days | 9 ^b^ |
|  |  |  | Adult | 2 Years | 2 |
|  |  |  | Newborn | 7–45 Days | 9 ^c^ |
|  | hFIX | TBG | Newborn | 4 Years | 2 ^d^ |
|  | hIDUA | TBG |  | 4 Years | 2 ^d^ |
|  | hLDLR | TBG | Adult | 2 Years | 2 |
|  | rhCG | TBG | Adult | 6 Months | 6 |
|  | rhLDLR | TBG | Adult | 2 Years | 2 |
| AAV9 | eGFP | CB7.CI | Adult | 3 Weeks | 6 |
| AAVhu37 | hFVIII | TBG-S1 | Adult | 3 Years | 1 |
|  |  | E12.A1AT | Adult | 1 Year | 5 ^e^ |
|  |  | ABP2.TBG-S1 | Adult | 6 Months | 2 |
| AAVrh10 | hFVIII | ABP2.TBG-S1 | Adult | 2 Years | 2 |
|  |  | E12.A1AT | Adult | 1 Year | 5 ^e^ |
|  |  | EnTTR.TTR | Adult | 1 Year | 5 ^e^ |
|  | rhCG | TBG | Adult | 6 Months | 6 |
| AAVrh64R1 | hFVIII | ABP2.TBG-S1 | Adult | 3 Years | 2 |

**^a^** *Rivera et al. Blood (2005). doi:10.1182/blood-2004-06-2501*

**^b^** *Greig et al. Molecular Therapy-Methods & Clinical Development (2016). doi: https://doi.org/10.1038/mtm.2016.79*

**^c^** *Wang et al., Molecular Therapy (2010). doi: https://doi.org/10.1038/mt.2009.245*

**^d^** *Hordeaux et al., Human Gene Therapy (2019). doi: 10.1089/hum.2019.012*

**^e^***Greig et al. Human Gene Therapy (2018). doi: https://doi.org/10.1089/hum.2018.080*
