# Supplementary material for: Prevalent and Disseminated Recombinant and Wild-Type Adeno-Associated Virus Integration in Macaques and Humans
Source: Hum Gene Ther. 2023 Nov 15;34(21-22):1081–94. doi: 10.1089/hum.2023.134 (PMC10659022; doi:10.1089/hum.2023.134)
Supplement: Supplemental data [file Supp_TableS2.docx]

**Table S2: Details of the categories of different human samples**

Avg: average; UIL: unique integration locus.

| **Health Status** | **# of Subjects** | **Sex** | **# with Detectable Integration** | **# with Detectable *AAVS1*+** | **Avg # UILs/100 Genomes** | **Avg % of Site-Specific UILs** | **Avg % of Clonal UILs** | **Average Clone Size** | **Largest Clone Detected** |
| --- | --- | --- | --- | --- | --- | --- | --- | --- | --- |
| Healthy | 26 | M (52%)  F (48%) | 14 (52%) | 14 (54%) | 0.0392 | 1.91% | 8.29% | 2.66 | 28 |
| Carcinoma | 15 | M (100%) | 8 (57%) | 8 (57%) | 0.0158 | 2.73% | 6.87% | 2.03 | 3 |
| Diseased | 44 | M (70%)  F (30%) | 29 (66%) | 29 (66%) | 0.0192 | 0.65% | 9.68% | 2.80 | 7 |
