# Supplementary material for: Prevalent and Disseminated Recombinant and Wild-Type Adeno-Associated Virus Integration in Macaques and Humans
Source: Hum Gene Ther. 2023 Nov 15;34(21-22):1081–94. doi: 10.1089/hum.2023.134 (PMC10659022; doi:10.1089/hum.2023.134)
Supplement: Supplemental data [file Supp_FigS1.docx]

**
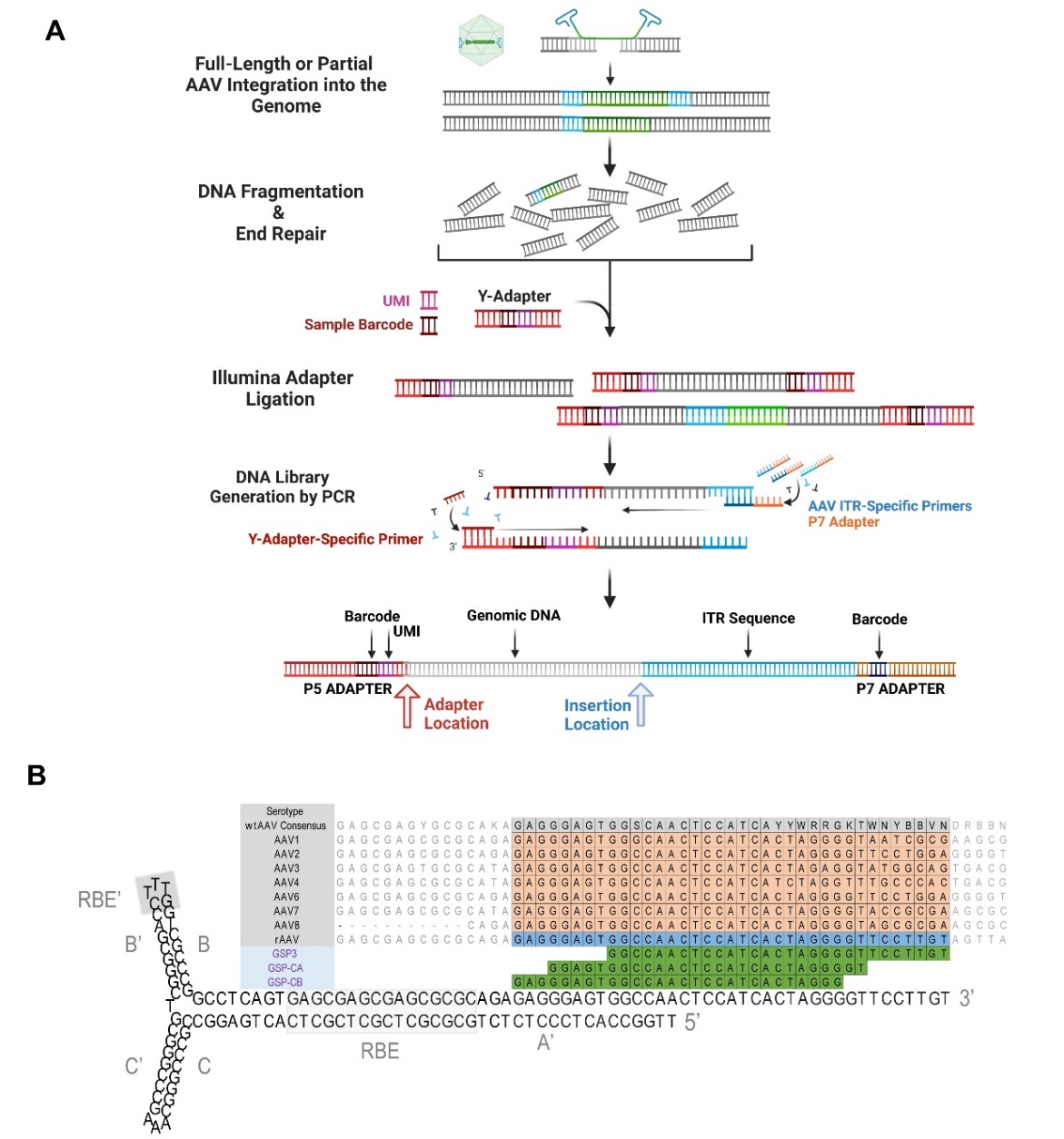
**

**Figure S1: Sample and library preparation**

(A) Illustration of next-generation sequencing library preparation. Using the tissue of interest, we isolate and shear the DNA into small fragments with an ultrasonicator. The DNA is purified and end-repaired, and Illumina Y-adapters are ligated. These Y-adapters contain two unique sequences: a sample barcode and a UMI, which is a unique 8-bp sequence that labels each DNA fragment to enable later removal of PCR duplicates. End-repaired Y-adapter-ligated DNA is amplified by PCR using an adapter-specific primer and the pool of primers specific for conserved regions of all known AAV ITR sequences. This approach produces NGS-compatible libraries with amplicons that contain both the amplified integrated AAV and the adjacent genomic DNA sequences. The DNA libraries are loaded onto an Illumina MiSeq sequencer, and 2x150-bp short-read sequencing is performed. AAV: adeno-associated virus; ITR: inverted terminal repeat; PCR: polymerase chain reaction; UMI: unique molecular identifier.

(B) A schematic showing the alignment of all known wtAAV ITR serotypes, the rAAV ITR sequence used, and the primers used in (A). The wtAAV consensus is the consensus sequence of all the serotypes. GSP: genome-specific primer; wtAAV: wild-type adeno-associated virus. See Breton *et al.* (2020). doi: 10.1186/s12864-020-6655-4.
