# Supplementary material for: Prevalent and Disseminated Recombinant and Wild-Type Adeno-Associated Virus Integration in Macaques and Humans
Source: Hum Gene Ther. 2023 Nov 15;34(21-22):1081–94. doi: 10.1089/hum.2023.134 (PMC10659022; doi:10.1089/hum.2023.134)
Supplement: Supplemental data [file Supp_FigS2.docx]

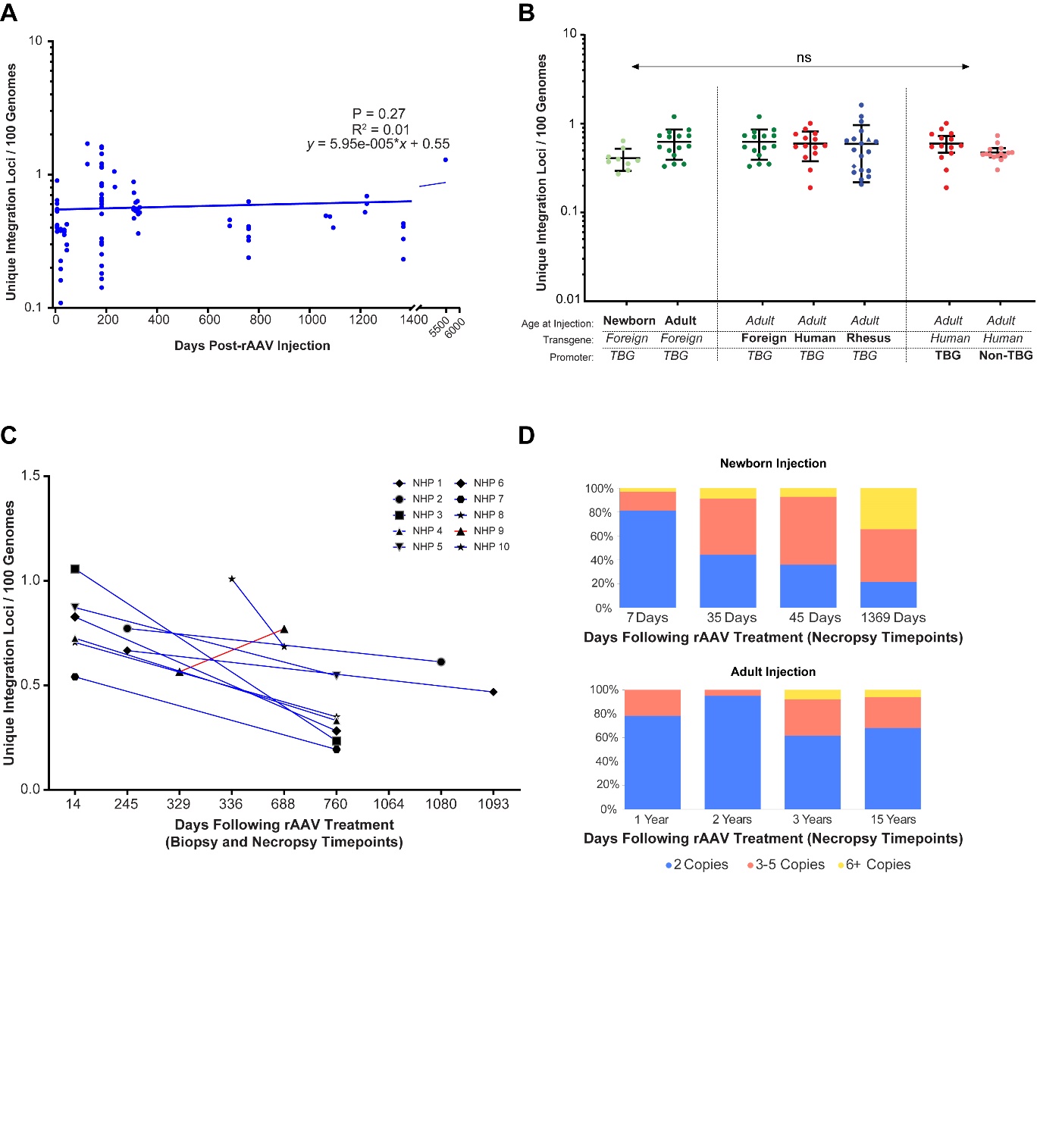
**Figure S2: Evaluation of variables potentially affecting rAAV-associated integration**

(A) Each point on the scatter plot represents an individual rAAV-treated nonhuman primate (NHP). The time of necropsy ranged from 7 days to 15 years after rAAV treatment. The number of unique AAV integration loci (UIL) per 100 genomes was normalized to a dose of 1×10^13^ GC/kg. rAAV: recombinant adeno-associated virus.

(B) In our cohort, NHPs were categorized by the following variables of the rAAV gene therapy that was administered: **1. Age at injection** (newborn or adult), **2. Transgene** (Foreign: Green Fluorescent Protein; Human: Factor VIII, Low Density Lipoprotein Receptor; or Rhesus: Chorionic Gonadotropin, Alpha Fetoprotein, Low Density Lipoprotein Receptor), **3. Promoter** (TBG [Thyroxine-binding globulin] or non-TBG (Alpha-1 Antitrypsin or Transthyretin). The number of UILs was determined for each NHP. ns: non-significant.

(C) In our rAAV-treated cohort, 10 NHPs had an early biopsy timepoint. In these samples, the timepoint of biopsy collection was less than 1 year after rAAV treatment, and the later necropsy timepoint was 2–3 years after rAAV treatment. Each individual NHP is represented by the same data point symbol with a line connecting the early biopsy timepoint and late necropsy timepoint. The number of UILs detected was compared between timepoints. Overall, 9/10 of the NHPs had fewer UILs detected at the necropsy timepoint compared with the biopsy timepoint. The result for the remaining NHP in which the number of UILs detected at necropsy was higher than at the biopsy timepoint is shown in red.

(D) In our cohort, all rAAV-treated newborn NHPs and all rAAV-treated adult NHPs were grouped by necropsy timepoint and analyzed to determine the proportion of clonal insertion loci with a given number of copies detected. Insertion sites with only one copy are considered non-clonal and are not represented in this graph. In each of the bars, blue represents loci with 2 copies, orange represents loci with 3–5 copies, and yellow represents loci with 6 or more copies detected.
