# Supplementary material for: Prevalent and Disseminated Recombinant and Wild-Type Adeno-Associated Virus Integration in Macaques and Humans
Source: Hum Gene Ther. 2023 Nov 15;34(21-22):1081–94. doi: 10.1089/hum.2023.134 (PMC10659022; doi:10.1089/hum.2023.134)
Supplement: Supplemental data [file Supp_FigS3.docx]

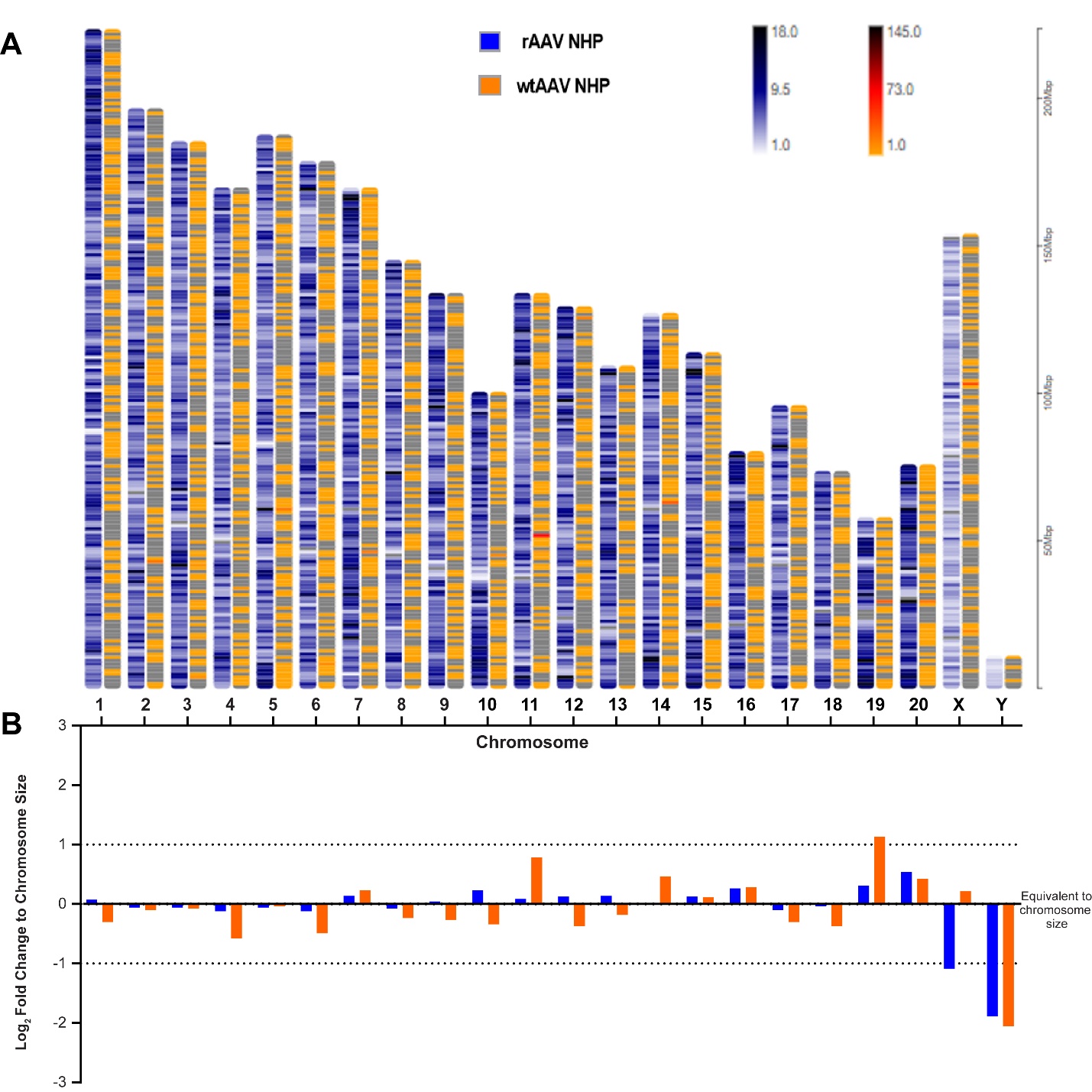


**Figure S3: Chromosomal distribution of wtAAV and rAAV integrations in the Rhesus Macaque genome**

The genomic locations of each unique AAV integration site for each rhesus macaque sample were mapped to their chromosomal location within their respective host genome (for rhesus we utilized reference genome MmuI_10). Detectable rAAV integration loci are on the left (blue), and wtAAV integration loci on the right (orange).

(A) The top graph shows a chromosome representation of locations of integration loci with heatmap coloring to indicate the number of copies detected at each locus. The chromosomes are grey in color. Maps were created in R using the ChromoMap package.

(B) The bottom graph shows the number of UILs detected in each chromosome compared with the expected number based on chromosome size. NHP: nonhuman primate; rAAV: recombinant adeno-associated virus; UIL: unique integration locus; wtAAV: wild-type adeno-associated virus.
