# Supplementary material for: Prevalent and Disseminated Recombinant and Wild-Type Adeno-Associated Virus Integration in Macaques and Humans
Source: Hum Gene Ther. 2023 Nov 15;34(21-22):1081–94. doi: 10.1089/hum.2023.134 (PMC10659022; doi:10.1089/hum.2023.134)
Supplement: Supplemental data [file Supp_FigS5.docx]

**
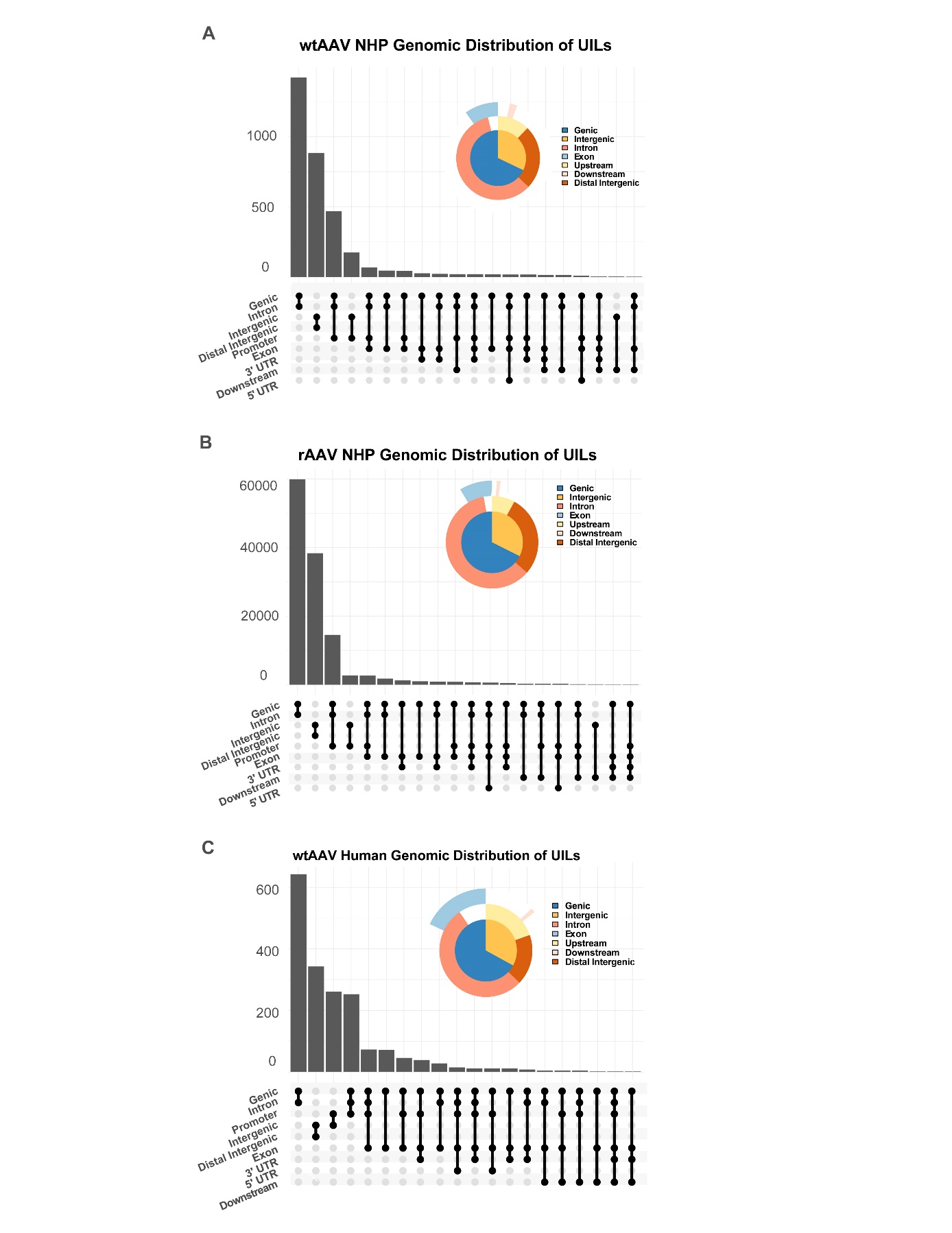
**

**Figure S5: Detailed genomic distribution of wtAAV and rAAV integrations**

(A–C) To annotate the location of a given locus in terms of genomic features, the ChIPseeker R package was used to annotate the following regions: TSS, exon, 5’ UTR, 3’ UTR, intronic, or intergenic. The Venn pie graph shows the distribution of loci within each category. The bar graph shows the distribution of the different intersections in a matrix. NHP: nonhuman primate; rAAV: recombinant adeno-associated virus; UIL: unique integration locus; UTR: untranslated region; wtAAV: wild-type adeno-associated virus.
