# Supplementary material for: Prevalent and Disseminated Recombinant and Wild-Type Adeno-Associated Virus Integration in Macaques and Humans
Source: Hum Gene Ther. 2023 Nov 15;34(21-22):1081–94. doi: 10.1089/hum.2023.134 (PMC10659022; doi:10.1089/hum.2023.134)
Supplement: Supplemental data [file Supp_FigS7.docx]

**
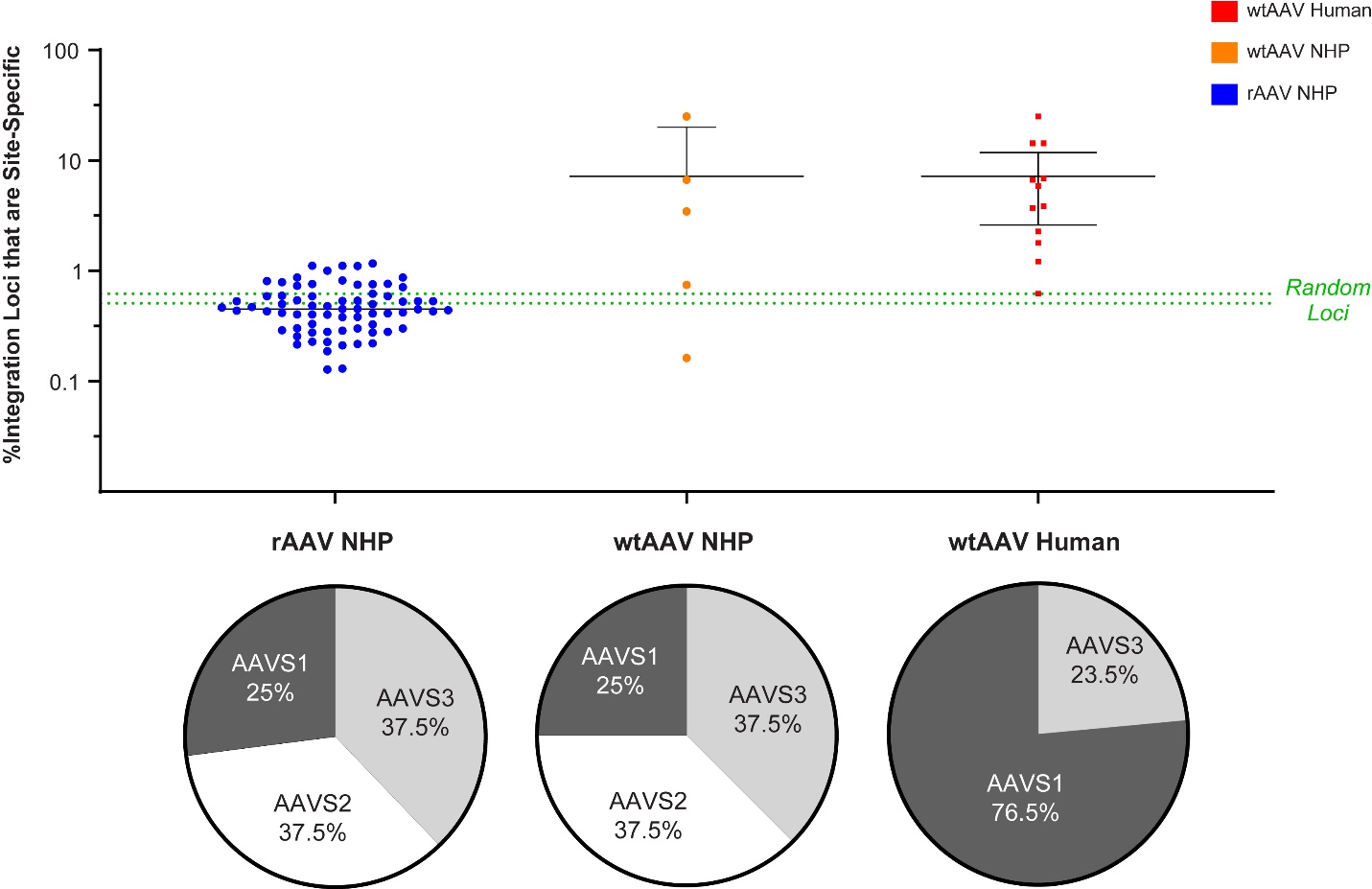
**

**Figure S7: Characterization of site-specific integrations**

The percentage of site-specific integration loci was determined for each sample. The dashed lines correspond to the range of random loci within a site-specific integration location within the 3 host genomes. Below each group is a pie chart representing the proportion of site-specific loci in each AAVS location. For each species, the coordinates used for each AAVS site are as follows: human (GRCh38.p13) AAVS1: chr19:53,100,001–55,800,000; AAVS2: chr5:28,900,001–33,800,000; AAVS3: chr3:16300001–23800000; rhesus (MmuI_10) AAVS1: chr19:52871888–55563490; AAVS2: chr6:29000422–33933291; AAVS3: chr2:23869443–31378156; cynomolgus (Macaca_fascicularis_5.0) AAVS1: chr19:53911962–56643542; AAVS2: chr6:29397402–34531455; AAVS3: chr2:23678813–31274157. NHP: nonhuman primate; rAAV: recombinant adeno-associated virus; wtAAV: wild-type adeno-associated virus.
